# Replication and shedding of MERS-CoV in Jamaican fruit bats (*Artibeus jamaicensis*)

**DOI:** 10.1038/srep21878

**Published:** 2016-02-22

**Authors:** Vincent J. Munster, Danielle R. Adney, Neeltje van Doremalen, Vienna R. Brown, Kerri L. Miazgowicz, Shauna Milne-Price, Trenton Bushmaker, Rebecca Rosenke, Dana Scott, Ann Hawkinson, Emmie de Wit, Tony Schountz, Richard A. Bowen

**Affiliations:** 1 Laboratory of Virology, Division of Intramural Research, National Institute of Allergy and Infectious Diseases, National Institutes of Health, Hamilton, Montana, USA; 2 Department of Biomedical Sciences, Colorado State University, Fort Collins, Colorado, USA; 3 Rocky Mountain Veterinary Branch, National Institute of Allergy and Infectious Diseases, National Institutes of Health, Hamilton, Montana, USA; 4 Department of Biology, University of Northern Colorado, Greeley, Colorado, USA; 5 Arthropod-borne and Infectious Diseases Laboratory, Department of Microbiology, Immunology and Pathology, Colorado State University, Fort Collins, Colorado, USA

## Abstract

The emergence of Middle East respiratory syndrome coronavirus (MERS-CoV) highlights the zoonotic potential of *Betacoronaviruses*. Investigations into the origin of MERS-CoV have focused on two potential reservoirs: bats and camels. Here, we investigated the role of bats as a potential reservoir for MERS-CoV. *In vitro*, the MERS-CoV spike glycoprotein interacted with Jamaican fruit bat (*Artibeus jamaicensis*) dipeptidyl peptidase 4 (DPP4) receptor and MERS-CoV replicated efficiently in Jamaican fruit bat cells, suggesting there is no restriction at the receptor or cellular level for MERS-CoV. To shed light on the intrinsic host-virus relationship, we inoculated 10 Jamaican fruit bats with MERS-CoV. Although all bats showed evidence of infection, none of the bats showed clinical signs of disease. Virus shedding was detected in the respiratory and intestinal tract for up to 9 days. MERS-CoV replicated transiently in the respiratory and, to a lesser extent, the intestinal tracts and internal organs; with limited histopathological changes observed only in the lungs. Analysis of the *innate* gene expression in the lungs showed a moderate, transient induction of expression. Our results indicate that MERS-CoV maintains the ability to replicate in bats without clinical signs of disease, supporting the general hypothesis of bats as ancestral reservoirs for MERS-CoV.

In 2012 a novel coronavirus, Middle East respiratory syndrome coronavirus (MERS-CoV), was isolated from a patient with a fatal case of pneumonia and renal failure in the Kingdom of Saudi Arabia^1^. Since then, more than 1600 human cases of MERS-CoV have been reported with a case-fatality rate of ~30%^2^. MERS-CoV cases were initially largely confined to six countries on the Arabian Peninsula: Jordan, Qatar, United Arab Emirates, Oman, Kuwait, and the majority of cases have been detected in the Kingdom of Saudi Arabia^2^. Travel-related cases have been identified in Europe, Asia, Africa and North America^3^. The recent outbreak caused by a single imported case in South Korea highlights the potential of MERS-CoV to cause large outbreaks when appropriate infection control measures are lacking in health care settings^3^,^4^.

MERS-CoV is classified within the group C *Betacoronaviruses*^4^,^5^. Since the emergence of MERS-CoV, considerable effort has been expended to identify potential natural and intermediate reservoir hosts. A variety of closely related coronavirus sequences have been obtained from numerous bat species in Africa, Asia, the Americas and Eurasia^6^,^7^,^8^,^9^,^10^,^11^,^12^,^13^,^14^,^15^,^16^,^17^,^18^,^19^, suggesting widespread distribution of MERS-CoV related viruses among bat species belonging to the order Chiroptera. A 190 nucleotide RNA fragment with 100% match to the RNA dependent RNA polymerase (RdRp) of MERS-CoV was detected in a fecal sample of an Egyptian tomb bat (*Taphozous perforates*) roosting in the vicinity of a MERS-CoV index case in Bisha, Kingdom of Saudi Arabia^20^. In addition, the detection of MERS-CoV-specific antibodies in dromedary camels in the Middle East and northern and eastern Africa suggest the widespread circulation of MERS-CoV-like viruses in dromedary camels^21^,^22^,^23^,^24^,^25^,^26^,^27^. MERS-CoV infection of dromedary camels was detected by qRT-PCR and virus isolation in the Kingdom of Saudi Arabia, Qatar, Oman, United Arab Emirates and Egypt^23^,^28^,^29^,^30^. MERS-CoV sequences and virus isolates obtained from dromedary camels in Qatar and the Kingdom of Saudi Arabia showed high sequence identity with those obtained from epidemiologically linked human cases^28^,^31^. Together, these data suggest that rather than direct zoonotic transmission from a bat reservoir, dromedary camels are involved as the primary reservoir host for MERS-CoV. Phylogenetic and epidemiological data suggest that rather than a single introduction in the human population, MERS-CoV appears to continue to be transmitted by multiple independent spillover events from dromedary camels^32^,^33^.

After the emergence of severe acute respiratory syndrome coronavirus (SARS-CoV) in 2003, the targeted focus on reservoir studies in bats has resulted in a vast increase of our knowledge on the genetic diversity of bat coronaviruses. Despite the increase in genetic data on coronavirus diversity in their natural reservoirs, only very limited data are available on the impact of these viruses on the reservoir host and controlled infection experiments with coronaviruses in their reservoir hosts have not been performed. To understand the drivers of MERS-CoV emergence, a more comprehensive understanding of the interaction between the virus and its natural and intermediate reservoir hosts is needed. Here we present data on the first experimental infection of bats with MERS-CoV to model the infection kinetics in a coronavirus host species, the Jamaican fruit bat (*Artibeus jamaicensis*).

## Results

### *Artibeus jamaicensis* DPP4 receptor and cell susceptibility

**Figure 1:**
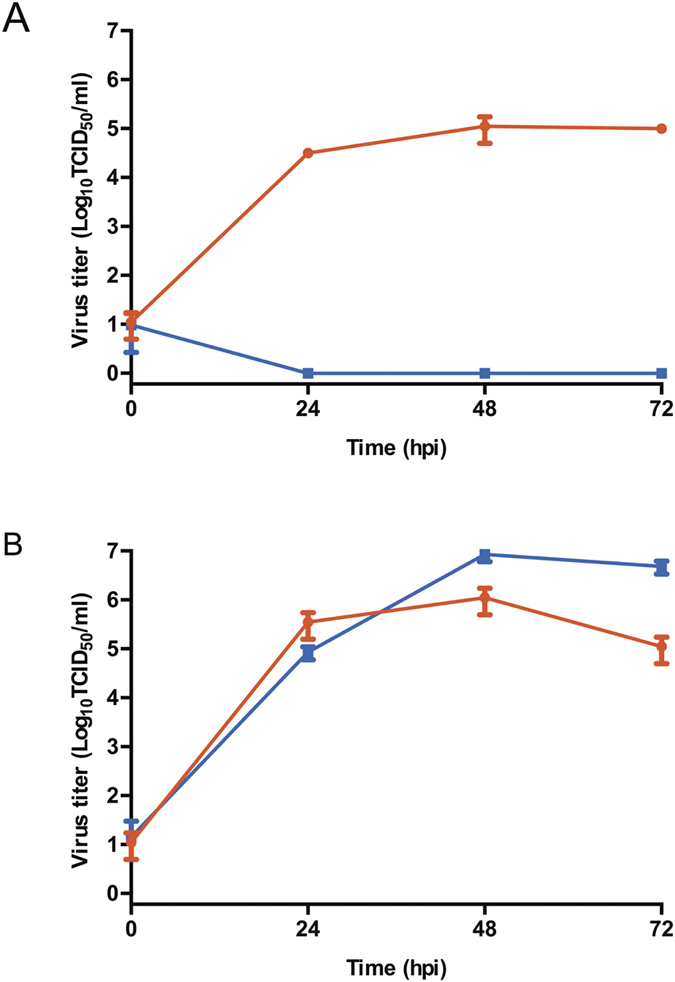
Replication kinetics of MERS-CoV in cells expressing bat DPP4. (**A**) Hamster BHK transfected to express Jamaican fruit bat (red) and hamster DPP4 (blue) and inoculated with MERS-CoV (MOI of 1 TCID_50_/cell). (**B**) Jamaican fruit bat primary kidney (red) and VeroE6 (blue) cell lines inoculated with MERS-CoV (MOI of 0.01 TCID_50_/cell). Supernatants were harvested at 0, 24, 48 and 72 hpi and viral titers were determined by end-point titration in quadruplicate in VeroE6 cells. Geometric mean titers were calculated from three independent experiments. Error bars indicate standard deviations.

The MERS-CoV receptor DPP4 is the main host restriction factor^34^; therefore, we first studied the interaction between MERS-CoV and Jamaican fruit bat DPP4. The pAJ-DPP4 plasmid expressing the DPP4 coding sequence of Jamaican fruit bat under control of a CMV promoter was transfected into BHK cells, which are not permissive to MERS-CoV^34^. The expression of AJ-DPP4 in transfected cells was confirmed by flow cytometry, showing the presence of bat DPP4 on the surface of transfected BHK cells by determining the increase over untransfected cells (Figure S1). Transient expression of bat DPP4 in BHK cells supported MERS-CoV replication, whereas transient expression of hamster DPP4 in BHK cells did not (Fig. 1A). Subsequently, the replication kinetics of MERS-CoV were compared in LLC-MK2 cells (*Macaca mulatta*) and Jamaican fruit bat primary kidney cells. MERS-CoV replicated efficiently to high titers in both cell lines (Fig. 1B), indicating that there is no restriction at the receptor or cellular level for MERS-CoV replication in Jamaican fruit bat cells.

### Clinical signs in bats inoculated with MERS-CoV

Ten adult Jamaican fruit bats were inoculated via the intranasal and intraperitoneal routes with 10^5^ TCID_50_ of MERS-CoV strain EMC/2012; 2 mock inoculated Jamaican fruit bats, housed in a separate cage, were used as controls, the mock inoculated animals were inoculated with tissue culture medium via the same routes and volumes. The bats were observed at least once daily for signs of disease. Bodyweight and temperature were measured throughout the experiment for a maximum of 28 days post inoculation (dpi) for bat 9 and 10 (MERS-CoV inoculated) and bat 11 and 12 (mock inoculated controls). None of the bats showed signs of disease, weight loss or increased body temperature throughout the experiment (Figure S2).

### MERS-CoV shedding in bats inoculated with MERS-CoV

**Figure 2:**
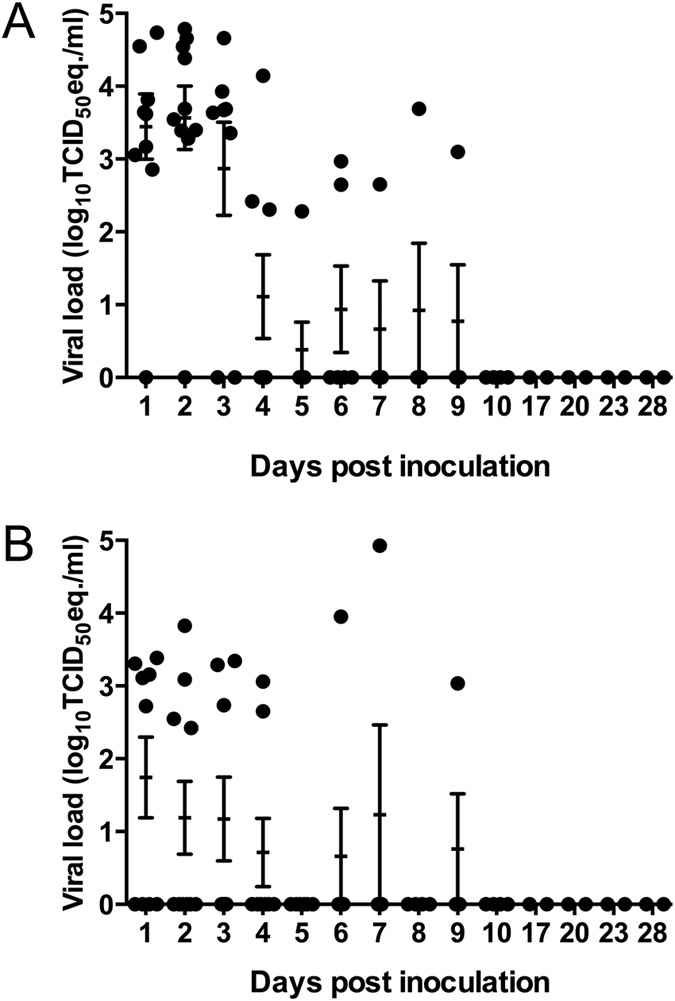
MERS-CoV shedding in Jamaican fruit bats. (**A**) from the respiratory tract of Jamaican fruit bats. (**B**) from the intestinal tract of Jamaican fruit bats. Oral and rectal swabs were collected at exam and necropsy dates. RNA was extracted and qRT-PCR was performed. Viral load was expressed as TCID_50_ equivalents.

To examine MERS-CoV shedding in inoculated bats, oral and rectal swabs were collected for the duration of the experiment. MERS-CoV shedding was first detected on 1 dpi, as indicated by the presence of viral RNA in throat and rectal swabs and continued for a maximum duration of 9 days. All animals, except bat 10, shed MERS-CoV from the respiratory tract (Fig. 2A); all bats except 4 and 10, shed MERS-CoV from the intestinal tract (Fig. 2B). Viral loads in swabs collected from the respiratory tract were higher than viral loads in swabs from the intestinal tract.

### MERS-CoV replication in Jamaican fruit bats

**Figure 3:**
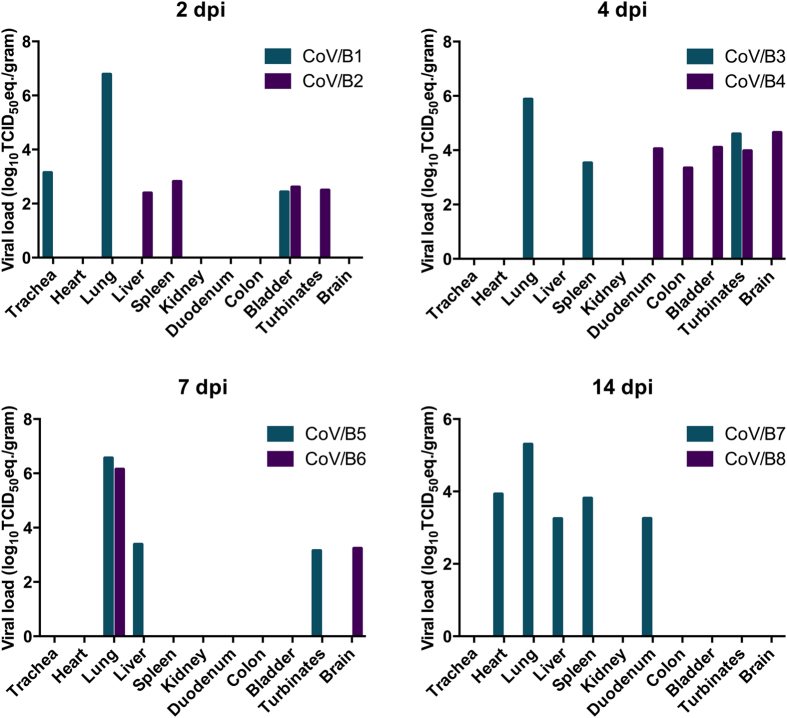
MERS-CoV viral load in tissues collected from Jamaican fruit bats inoculated with MERS-CoV. Tissues were collected at necropsy on the indicated days post-inoculation. RNA was extracted and qRT-PCR was performed. Viral load was expressed as TCID_50_ equivalents per gram tissue.

Tissues collected at the sequential necropsy dates of 2, 4, 7, 14 and 28 dpi were analyzed for the presence of viral RNA, infectious virus, and evaluated by histopathology and immunohistochemistry. MERS-CoV viral RNA was detected in various tissues of all inoculated bats, except bat 8 on 14 dpi and bat 9 on 28 dpi. The highest viral loads were detected in the lower respiratory tract (Fig. 3). MERS-CoV viral RNA was detected at 2 dpi in trachea, lung, liver, spleen, bladder and nasal turbinates; at 4 dpi in lung, spleen, duodenum, colon, bladder, turbinates and brain; at 7 dpi in lung, liver, turbinates and brain; at 14 dpi in heart, lung, liver, spleen and duodenum. No MERS-CoV viral RNA was detected at 28 dpi (Fig. 3). In additon, MERS-CoV viral RNA was detected in blood on 2 and 4 dpi, in bats 1-3, indicative of viremia (Figure S3). MERS-CoV mRNA was detected in tissues of bats 1 to 7, confirming MERS-CoV replication on the transcriptional level (Table S2).

Infectious MERS-CoV was isolated from the lungs of bats 1 (2 dpi) and 6 (7 dpi), the bladder and nasal turbinates of bat 7 (14 dpi), and the duodenum of bat 10 (28 dpi), indicating active virus replication, mainly in the respiratory tract.

**Figure 4:**
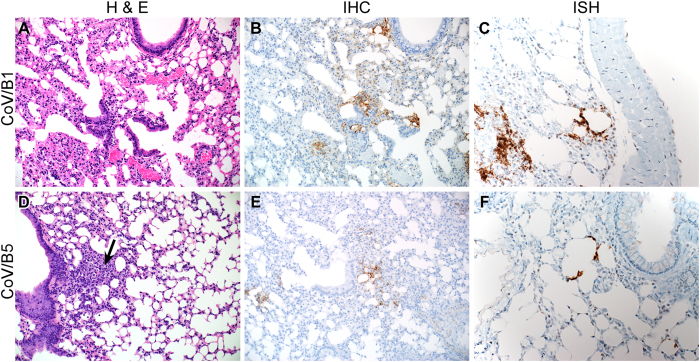
Histopathological changes in Jamaican fruit bats inoculated with MERS-CoV. Jamaican fruit bats were euthanized on day 2, 4, 7, 14 and 28 days post inoculation and tissue was collected, formalin fixed and stained with hematoxylin and eosin (H&E; panels **A,C**) or immunohistochemistry using a polyclonal α-MERS-CoV antibody (IHC; panels **B,D**). Shown are bat 1 (panel **A–C**) and bat 5 (panel **D–F**). (**A**) Bat 1, no pulmonary pathology detected on 2 dpi visible is apparently normal lungtissue. (**B**) IHC staining of sequential section of panel A reveals abundance of MERS-CoV antigen in bat 1. (**C**) ISH staining of sequential section of panel (**A**) reveals abundance of MERS-CoV RNA in bat 1. (**D**) Bat 5, minimal alveolar thickening with infiltration of small numbers of foamy macrophages and fewer neutrophils (indicated by black arrow). (**E**) IHC staining of sequential section of panel C reveals abundance of MERS-CoV antigen in affected areas in bat 5. (**F**) ISH staining of sequential section of panel A reveals abundance of MERS-CoV RNA in bat 5. Original magnification: 20× for (**A, B, D**) and (**E**) and 40× for (**C**) and (**F**).

**Figure 5:**
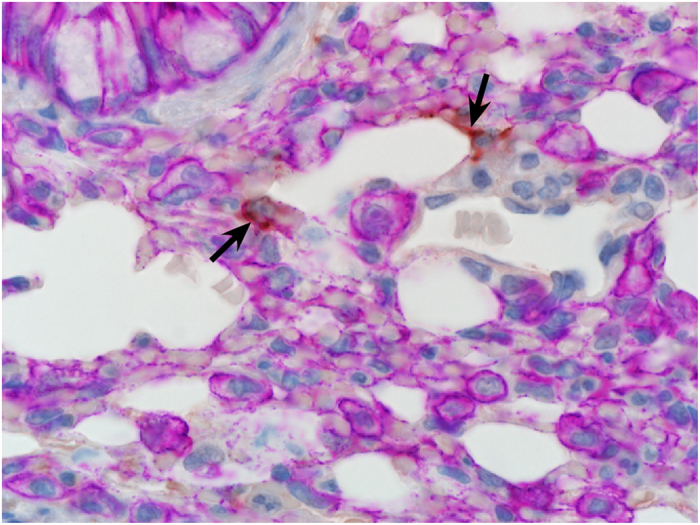
MERS-CoV replicates in alveolar pneumocytes. Lungs of Jamaican fruit bat 5 were stained with α-cytokeratin as an epithelial marker (purple) and with a polyclonal α-coronavirus antibody (brown-red) to demonstrate that viral antigen was located along the basement membrane of alveolar pneumocytes of bat 1 at 2 dpi (indicated by black arrows). Original magnification: 40×.

Only two of ten bats (bat 3 and bat 5) exhibited histopathology associated with MERS-CoV infection, which was mild. All MERS-CoV associated lesions were detected in the respiratory tract of the infected bats (Fig. 4, Table S1). Bat 3 and bat 4 (4 dpi) displayed a mild acute rhinitis, but MERS-CoV replication was not detected by immunohistochemistry (Table S1 and S2). Bat 3 and 5 displayed a multifocal interstitial pneumonia that was characterized by minimal alveolar interstitial thickening by small numbers of macrophages and neutrophils (Fig. 4, Table S1). The adjacent alveolar spaces contained small numbers of alveolar macrophages. MERS-CoV antigen and RNA was detected by immunothroughout the lungs of bat 1 (2 dpi), but no associated pulmonary pathology was detected (Fig. 4, Table S2). Cytokeratin and anti-MERS-CoV co-staining demonstrated MERS-CoV antigen in type I pneumocytes of the lungs of bat 1 (Fig. 5).

### Innate immune response to MERS-CoV

**Figure 6:**
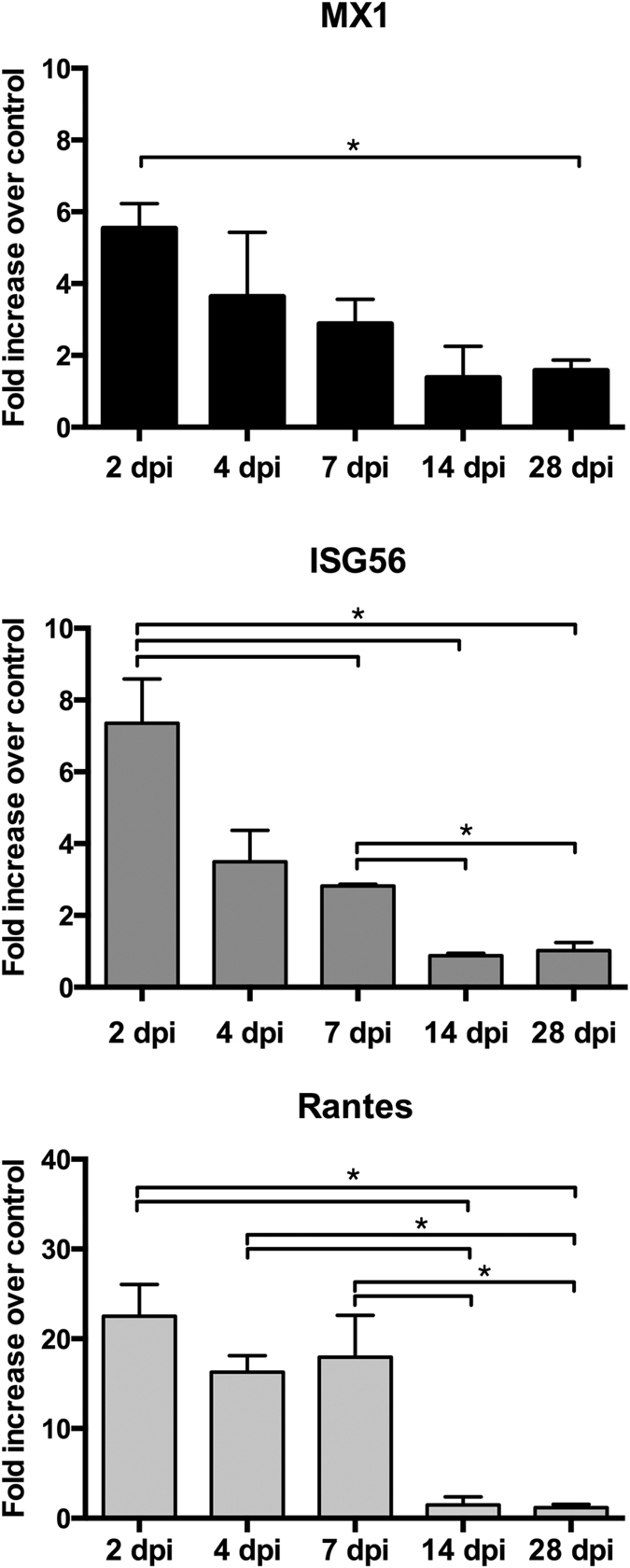
*Mx1, ISG56 and RANTES* upregulation after MERS-CoV infection in Jamaican fruit bats. Gene expression was determined by qRT-PCR and normalized using HPRT expression. Data are the mean and standard error of mean calculated from two Jamaican fruit bats at each time point and is represented as fold increase over uninfected controls. Statistical significance was calculated using two-tailed unpaired *t*-tests.

MERS-CoV was most consistently detected in the lower respiratory tract of the bats. The *Mx1, ISG56 and RANTES* gene expression in the lungs of Jamaican fruit bats was analyzed as an indicator of the induction of an innate immune response to MERS-CoV infection. A 6-fold increase in expression of *Mx1*was observed in the lungs of the infected Jamaican fruit bats at 2 dpi. A statistically significant upregulation of *Mx1* gene expression was detected when comparing the lungs of bats collected on 2 dpi and 28 dpi (two-tailed unpaired *t*-tests, *p* < 0.034). The maximum *ISG56* expression of 7.4-fold occurred at 2 dpi. Statistically significant differences were observed between the 2 dpi and 7 dpi, 14 dpi and 28 dpi animals (two-tailed unpaired *t*-tests, *p* < 0.035, p < 0.0178 and p < 0.0192 respectively. In addition significant differences were observed between the 7 dpi and the 14 and 28 dpi animals (two-tailed unpaired *t*-tests, *p* < 0.0009 and p < 0.0085). The RANTES expression at its peak at 2 dpi was increased 22.5 fold. Statistically significant differences were observed between the 2 versus the 14 and 28 dpi animals (two-tailed unpaired *t*-tests, *p* < 0.0147 and p < 0.0136), the 4 versus the 14 and 28 dpi animals (two-tailed unpaired *t*-tests, *p* < 0.0092 and p < 0.0075), and the 7 dpi versus the 14 and 28 dpi animals (two-tailed unpaired *t*-tests, *p* < 0.0390 and p < 0.0366) (Fig. 6).

### Antibody response to MERS-CoV

Sera were collected prior to inoculation and at the scheduled necropsy dates. Each of the bats was seronegative for MERS-CoV prior to inoculation. Only bat 7 developed a MERS-CoV specific antibody response, both by ELISA and virus neutralization assay. The sera obtained from bat 7 had a neutralizing titer of 320 at 14 days post inoculation.

## Discussion

The high sequence similarity of MERS-CoV to coronavirus sequences detected in bats suggests that MERS-CoV or its immediate ancestor originated in bats^35^. Direct contact between bats and humans is uncommon, and a domestic or peridomestic intermediate species often plays a role in the emergence of zoonotic viruses from natural reservoirs to humans^36^,^37^,^38^,^39^. Similar to the emergence of SARS-CoV in 2002 from the masked palm civet (*Paguma larvata*) as an intermediate host^40^, the dromedary camel appears to have initially served as the intermediate host for MERS-CoV^41^. Several aspects of the emergence of MERS-CoV are currently still unknown, including the role of the natural reservoir and the relationship between the natural and intermediate reservoirs.

*Artibeus spp.* bats inhabit a large geographical area throughout the tropical North and South Americas^42^. A wide variety of alpha- and betacoronaviruses have been detected in *Artibeus spp.* bats in Costa Rica, Ecuador, Mexico, Panama and Trinidad & Tobago^7^,^13^,^43^,^44^. The wide diversity of coronaviruses detected in bats, combined with their ability to support efficient replication of MERS-CoV, the availability of an annotated transcriptome^45^, and the relative easy housing and husbandry practices of Jamaican fruit bats suggest that this bat species can become an important model system to investigate the relationship between coronaviruses and their bat hosts^46^. Although the Jamaican fruit bat is not the direct ancestral reservoir for MERS-CoV, as it is a new world bat species, generalized responses towards viruses of bat-origin rather than a direct host-pathogen relationships can be modelled.

The ability of MERS-CoV to use DPP4 of multiple species as a receptor, including DPP4 of human, dromedary camel, and bat origin^34^,^47^, suggests that no prior adaptation was needed on the DPP4 receptor level for cross-species and zoonotic transmission to occur. With batCoV-HKU4, a closely related coronavirus, it was shown that replication in human cells required two mutations in the spike protein^48^. These amino acid residues, which are conserved in MERS-CoV, results in the activation of the batCoV-HKU4 spike protein by human cellular proteases. This suggests that batCoV-HKU4 needs these residues for replication in humans. Interestingly, our data show that MERS-CoV replicates efficiently in Jamaican fruit bat cells, suggesting that the MERS-CoV spike can efficiently be processed by Jamaican fruit bat cellular proteases and that there is no host restriction on the post-translational modification level of the MERS-CoV spike in dromedary camels, humans and bats.

Bat coronaviruses have been primarily detected in fecal samples in field studies suggesting that these viruses have a intestinal tract tropism^9^,^19^,^43^. MERS-CoV was able to replicate to higher titers in the respiratory tract in comparison with the intestinal tract of the Jamaican fruit bats. The tissue tropism of MERS-CoV in Jamaican fruit bats is comparable to the respiratory tract tropism observed in dromedary camels and humans^49^,^50^. This might suggest that MERS-CoV, upon cross-species transmission from bats into dromedary camels evolved from a gastrointestinal tract virus into a respiratory tract virus, similar to influenza A viruses^51^.

The ability for MERS-CoV to antagonize the innate immune response appears to correlate with its pathogenic potential in humans. MERS-CoV and related batCoV-HKU4 can inhibit innate immune signaling in a variety of human cell lines *in vitro* via the ORF4b-encoded accessory proteins^52^. The ability of MERS-CoV and batCoV-HKU4 to evade this innate immune sensing must have evolved in the reservoir bat host, and may therefore have benefitted the virus by limiting the bat immune response without negatively impacting the population level of the bat host. The modest, transient upregulation of *Mx1, ISG56 and RANTES* observed in Jamaican fruit bats may permit MERS-CoV replication without causing clinical signs. The transient nature of the MERS-CoV infection suggests that the virus is cleared by immune mechanisms, but the isolation of MERS-CoV in the duodenum of bat 10 at 28 dpi indicates that the virus might persist at low levels for a longer period of time. The limited seroconversion observed in the Jamaican fruit bats suggest that the immunogenicity of the MERS-CoV proteins in the context of experimental infection in bats in absence of clinical signs is very low. In humans seroconversion was observed in clinically ill patients between 21 and 28 days after infection with MERS-CoV with titers between 80 and 320^53^. Limited seroprevalance was observed in healthy people with a high occupational risk for MERS-CoV exposure (shepherds and slaughterhouse workers), combined with the extensive circulation of MERS-CoV in dromedary camels this suggests that unrecognized infections that cause no or mild symptoms might result in no to limited seroconversion^22^,^54^.

Although of considerable interest, the number of experimental infection studies in bats are limited. Experimental bat infection studies have been performed with coronavirus bat intestine samples derived from field studies^18^, Nipah virus and Hendra virus^55^,^56^, Tacaribe virus^57^, Ebola virus and Marburg virus^58^,^59^ and the majority of experimental infection studies have been performed with Lyssaviruses^60^. In these studies replication, shedding, morbidity and mortality varied greatly. Severe morbidity and mortality were observed after inoculation of Jamaican fruit bats with Tacaribe virus and rabies virus^57^,^61^; Jamaican fruit bats are the presumed host reservoir for both viruses. After oral inoculation of Leschenault’s Rousette bats (*Rousettus leschenaulti)* with homogenates of intestinal tract samples obtained from bats caught during field studies in the Philippines and positive by PCR for a group 2 *Betacoronavirus* (closely related to MERS-CoV) no clinical signs were observed, but replication and limited virus shedding were observed in and from the intestinal tract^18^. Our experimental infection data show that MERS-CoV maintains the ability to efficiently replicate in a bat host. In our bat infection model all Jamaican fruit bats became infected with MERS-CoV in the absence of clinical signs. This supports the hypothesis that bats are the ancestral reservoir for MERS-CoV. In addition, it highlights the host-range plasticity of MERS-CoV, and potentially of those of highly related coronaviruses circulating in bat population.

## Materials and Methods

### Ethics Statement

All animal experiments were approved by the Institutional Animal Care and Use Committee of Colorado State University, and performed following the guidelines of the Association for Assessment and Accreditation of Laboratory Animal Care, International (AAALAC) in an AAALAC-approved facility. All work with MERS-CoV was performed under BSL3 containment at Colorado State University or the Rocky Mountain Laboratories, Division of Intramural Research, National Institute of Allergy and Infectious Diseases, National Institutes of Health and was approved by their respective Institutional Biosafety Committees.

### Virus and cells

MERS-CoV strain EMC/2012 was kindly provided by the Viroscience Laboratory, Erasmus Medical Center, Rotterdam, The Netherlands and propagated in Vero E6 cells in DMEM (Sigma) supplemented with 2% fetal calf serum (Hyclone, Logan), 1 mM L-glutamine (Lonza), 50 U/ml penicillin and 50 μg/ml streptomycin (Gibco). Vero E6, LLC-MK2, BHK and Jamaican fruit bat primary kidney cells were maintained in Dulbecco’s modified Eagle’s media (DMEM) supplemented with 10% fetal calf serum, 50 U/ml penicillin and 50 μg/ml of streptomycin.

### Sequencing and cloning of the Jamaican fruit bat DPP4 sequence

Total RNA was extracted from primary kidney cells using the RNeasy Mini Kit (Qiagen) and cDNA was synthesized using random hexamer primers and SuperScript III Reverse Transcriptase (Applied Biosystems). DPP4 was then amplified using iProof High-Fidelity DNA Polymerase (BioRad) and primers DPP4UnvF1 and DPP4UnvR12 (primer sequences are available upon request). The obtained DPP4 gene sequence was synthesized in expression plasmid pcDNA3.1(+) (GeneArt): pAJ-DPP4. The Jamaican fruit bat DPP4 nucleotide sequence is available in GenBank under accession number KF574262.

### Expression of DPP4

BHK cells were transiently transfected with 3 μg pAJ-DPP4 using 8 μl of Lipofectamine 2000 (Life Technologies). Expression of DPP4 was confirmed by flow cytometry as described previously^34^.

### Replication kinetics

Multistep replication kinetics were determined by inoculating wells of cells in triplicate with MERS-CoV with a multiplicity of infection (MOI) of 0.01 (cell lines) or 1 (transfected cell lines) 50% tissue culture infectious dose (TCID_50_) per cell. One hour after inoculation, cells were washed once with DMEM and culture medium replaced. Supernatants were sampled at 0, 24, 48 and 72 h after inoculation. MERS-CoV was titrated by end-point titration in quadruplicate in Vero E6 cells cultured in DMEM supplemented with 2% fetal calf serum, 1 mM L-glutamine (Lonza), 50 U/ml penicillin and 50 μg/ml streptomycin. Cells were inoculated with ten-fold serial dilutions of virus, and scored for cytopathic effect 5 days later. The TCID_50 _was calculated by the method of Spearman-Karber.

### Animal experiments

Twelve captive-bred Jamaican fruit bats were used for this work^46^,^57^. Ten bats were inoculated with 10^5^ TCID_50_ EMC/2012 via a combination of intranasal (25 μl each nostril) and intraperitoneal (100 μl) routes. Two mock inoculated bats were included as controls for histopathology and gene expression analyses. Mock inoculated bats were inoculated with standard tissue culture media via the same routes and volumes. Bats were injected with an IPTT-300 temperature transponder (BMDS) to monitor body temperature daily. Animals were weighed daily and observed for signs of disease. Oropharyngeal and rectal swabs were obtained on 1, 2, 3, 4, 5, 6, 7, 9 and 11 dpi and analyzed for the presence of viral RNA. On 2, 4, 7, 14 and 28 days post inoculation (dpi), two bats were euthanized and trachea, heart, lung, liver, spleen, kidney, duodenum, colon, bladder, nasal turbinates and brain were collected for virological and histopathological analysis.

### Histopathology

Histopathology was performed on bat tissues. After fixation for at least 7 days in 10% neutral-buffered formalin and embedding in paraffin, tissue sections were stained with hematoxylin and eosin (H & E) staining. Immunohistochemistry was performed using a MERS-CoV EMC/2012 polyclonal rabbit antibody at a 1:1000 dilution and in situ hybridization was performed using probes directed against the MERS-CoV EMC/2012 N gene as described previously^62^.

### RNA extraction

RNA was extracted from swab samples using the QiaAmp Viral RNA kit (Qiagen). RNA was eluted in 60 μl. Tissues (30 mg) were homogenized in RLT buffer and RNA was extracted using the RNeasy kit (Qiagen). RNA was eluted in 50 μl.

### Quantitative PCR

For detection of viral RNA in samples, 5 μl RNA was used in a one-step real-time RT-PCR upE assay^63^ using the Rotor-Gene^TM^ probe kit (Qiagen) according to the manufacturer instructions. In each run, standard dilutions of a titered MERS-CoV stock were run in parallel, to calculate TCID_50_ equivalents in the samples. For the detection of viral mRNA, 5 μl RNA was used in a one-step real-time RT-PCR using the MERS-CoV M mRNA assay in the Rotor-Gene^TM^ probe kit^64^. *Artibeus jamaicensis* orthomyxovirus resistance gene 1 (*Mx1*) gene expression was determined by qRT-PCR using *Mx1, ISG56* and *RANTES* specific primers (derived from transcriptome sequencing^45^). The fold-change of each gene was calculated by normalizing the change in CT (cycle threshold) value of *Mx1* (ΔCT) to the CT values for hypoxanthine phosphoribosyltransferase (HPRT) as an internal reference gene for each sample and comparing this to the CT values of mock inoculated bats 11 and 12 (2^∧^(−ΔΔCT). *Mx1* specific primers: 5′-CCAGACCTGACCCTGATAGA-3′, 5′-TGGATGTACTTCCTGAATGAGTTG-3′ and 5′-FAM-ATCTAGTGTCCGATGTCAGCTGGC-IABkFQ-3′. ISG56 specific primers: 5′- GCTGTCTATCGTCTGAATGGG-3′, 5′-TTCTTGTCCGATGTCCTGAAG-3′ and 5′-HEX- CGATGAGGC/Zen/ATTTTGTCTGCAAACCC-IABkFQ-3′. RANTES specific primers: 5′-AGTTGTCCTAATCACCCGAAAG-3′, 5′-CAGAGTGTTGATGTAGTCCCG-3′ and 5′-FAM-TGTGCCGAC/Zen/CCGGAGAAGAAAT-IABkFQ-3′. HPRT specific primers: 5′- AGATGGTGAAGGTCGCAAG-3′, 5′- CCTGAAGTATTCATTATAGTCAAGGG-3′ and 5′- FAM-ACTTTGTTGGATTTGAAATTCCAGACAAGTTTG-BHQ1.

### Virus isolation

Tissue samples were homogenized in a TissueLyzer II (Qiagen) after addition of 1ml DMEM. Homogenates were centrifuged to pellet cellular debris and subsequently inoculated onto VeroE6 and LLC-MK2 cells. After 1hr adsorption, cells were washed once with DMEM and media was replaced.

### ELISA

Antibody responses were measured in an enzyme-linked immunosorbent assay (ELISA) using hCoV-EMC/2012 as described previously^65^. Briefly, EMC/2012 containing cell culture supernatant was used to coat immuno 96 microwell maxisorp plates (NUNC) at 4 °C overnight and diluted serum samples were added. Bound antibodies were detected using a secondary protein A/G conjugated with horseradish peroxidase (HRP; Pierce). Sera were considered positive when absorbance was higher than three standard deviations above the mean of negative control sera. Sera obtained from rabbits immunized with EMC/2012 were used as a positive control.

### Virus Neutralization Assay

Two-fold serial dilutions of heat-inactivated sera were prepared in a 96 microwell tissue culture plate and 100 TCID_50_ of MERS-CoV was added and incubated for 1 hour at 37 °C. After incubation the virus-sera mixture was transferred to a 96 microwell tissue culture plate with a 95% confluent monolayer of VeroE6 cells. The virus neutralization titer was expressed as the reciprocal value of the highest dilution of the serum, that still inhibited EMC/2012 virus replication.

## Additional Information

**How to cite this article**: Munster, V. J. *et al.* Replication and shedding of MERS-CoV in Jamaican fruit bats *(Artibeus jamaicensis).* *Sci. Rep.* **6**, 21878; doi: 10.1038/srep21878 (2016).

## Supplementary Material

Supplementary Information
