# Supplementary material for: Replication and shedding of MERS-CoV in Jamaican fruit bats (Artibeus jamaicensis)
Source: Sci Rep. 2016 Feb 22;6:21878. doi: 10.1038/srep21878 (PMC4761889; doi:10.1038/srep21878)
Supplement: Supplementary Information [file srep21878-s1.pdf]

Supplemental information

Replication and shedding of MERS-CoV in Jamaican fruit bats (*Artibeus jamaicensis*)

Vincent J. Munster, Danielle R. Adney, Neeltje van Doremalen, Vienna R. Brown, Kerri L. Miazgowicz, Shauna Milne-Price, Trenton Bushmaker, Rebecca Rosenke, Dana Scott, Ann Hawkinson, Emmie de Wit, Tony Schountz and Richard A. Bowen

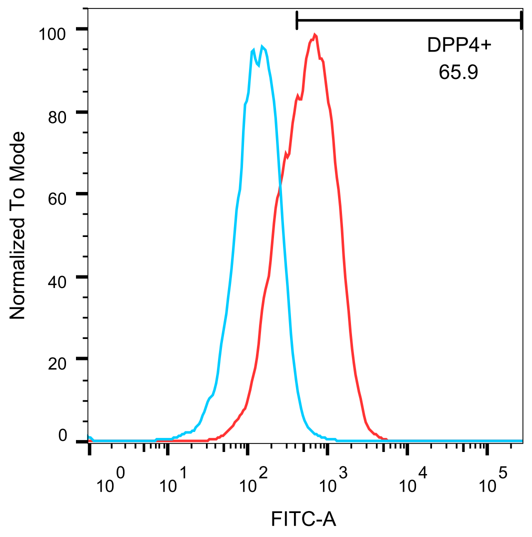

Figure S1. Surface expression of *Artibeus* bat DPP4 on BHK cells. Untransfected BHK cells (blue) or transfected with *Artibeus* DPP4 (red) and stained 24 h posttransfection using anti-DPP4 (R&D) and a FITC-conjugated secondary antibody (Life Technologies). Samples were collected using an LSRII flow cytometer (BD Biosciences) and analyzed using FlowJo software.

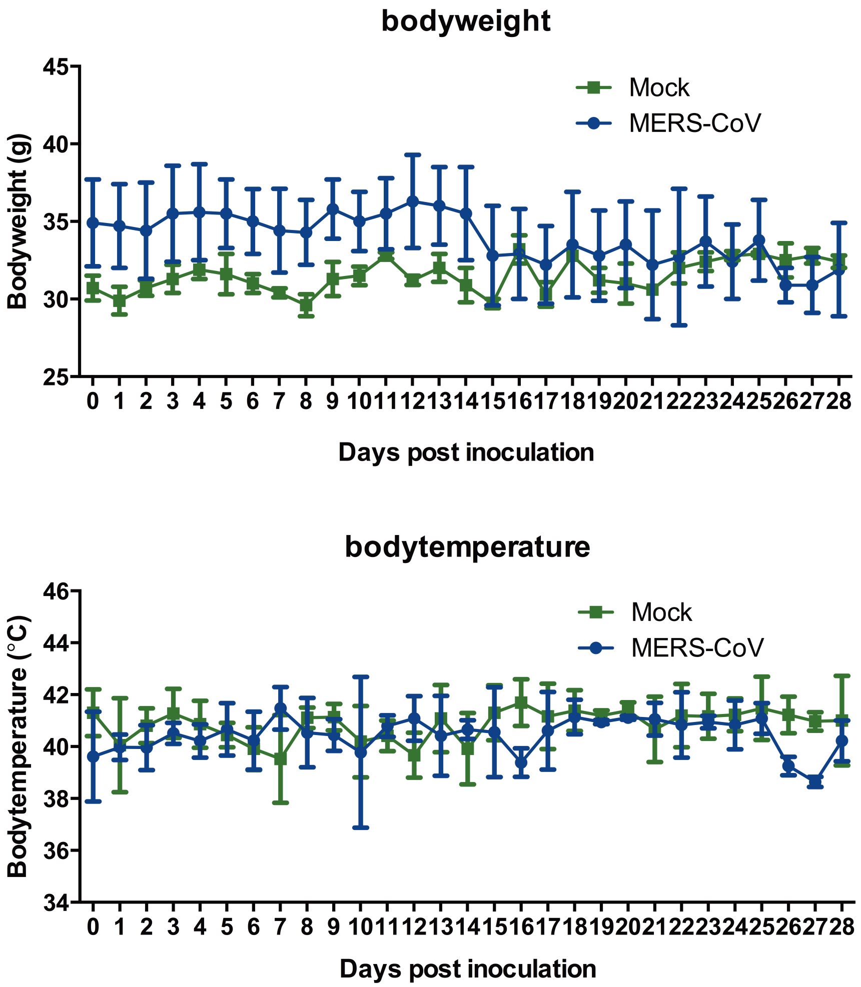

Figure S2. Bodyweight and temperature of MERS-CoV inoculated and mock inoculated control bats.

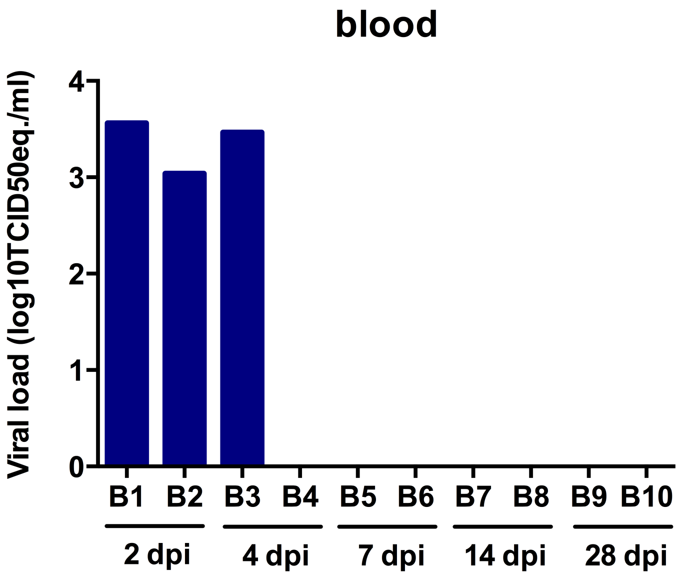

Figure S3. Detection of MERS-CoV viremia in Artibeus bats

Table S1. Summary of histopathological data

	H&E		IHC
	tissue	lesion	positive tissue
CoV/B1	-		lung
CoV/B2	-		-
CoV/B3	lung, nasal cavity	interstitial pneumonia, rhinitis	lung
CoV/B4	nasal cavity	rhinitis	-
CoV/B5	lung	interstitial pneumonia	lung
CoV/B6	-		-
CoV/B7	-		-
CoV/B8	-		-
CoV/B9	-		-
CoV/B10	-		-

Table S2. Summary of MERS-CoV data

	Evidence for MERS-CoV replication in Jamaican fruit bats						
	swabs (PCR)	tissue (PCR)	tissue (mRNA)	blood (PCR)	tissue (virus isolation)	histology (IHC/ISH)	seroconversion (ELISA & VN)
CoV/B1	+	+	+	+	+	+	
CoV/B2	+	+	+	+			
CoV/B3	+	+	+	+		+	
CoV/B4	+	+	+				
CoV/B5	+	+	+			+	
CoV/B6	+	+	+		+		
CoV/B7	+	+	+		+		+
CoV/B8	+						
CoV/B9	+						
CoV/B10					+		
